# Stefin B Inhibits NLRP3 Inflammasome Activation via AMPK/mTOR Signalling

**DOI:** 10.3390/cells12232731

**Published:** 2023-11-29

**Authors:** Mojca Trstenjak-Prebanda, Monika Biasizzo, Klemen Dolinar, Sergej Pirkmajer, Boris Turk, Veronique Brault, Yann Herault, Nataša Kopitar-Jerala

**Affiliations:** 1 Department of Biochemistry, Molecular and Structural Biology, Jožef Stefan Institute, SI-1000 Ljubljana, Slovenia; 2 International Postgraduate School Jožef Stefan, SI-1000 Ljubljana, Slovenia; 3 Institute of Pathophysiology, Faculty of Medicine, University of Ljubljana, SI-1000 Ljubljana, Slovenia; klemen.dolinar@mf.uni-lj.si (K.D.); sergej.pirkmajer@mf.uni-lj.si (S.P.); 4 Faculty of Chemistry and Chemical Technology, University of Ljubljana, SI-1000 Ljubljana, Slovenia; 5 Institut de Génétique et de Biologie Moléculaire et Cellulaire (IGBMC), INSERM, CNRS, Université de Strasbourg, 1 rue Laurent Fries, 67404 Illkirch Graffenstaden, France; vbrault@igbmc.fr (V.B.);; 6 Institut Clinique de la Souris, PHENOMIN, CELPHEDIA, INSERM, CNRS, Universite’ de Strasbourg, 67404 Illkirch Graffenstaden, France

**Keywords:** autophagy, AMPK, cystatin, EPM1, inflammasome, mitochondrial ROS, oxidative phosphorylation, mTOR signalling, stefin B

## Abstract

Stefin B (cystatin B) is an inhibitor of lysosomal and nuclear cysteine cathepsins. The gene for stefin B is located on human chromosome 21 and its expression is upregulated in the brains of individuals with Down syndrome. Biallelic loss-of-function mutations in the stefin B gene lead to Unverricht–Lundborg disease-progressive myoclonus epilepsy type 1 (EPM1) in humans. In our past study, we demonstrated that mice lacking stefin B were significantly more sensitive to sepsis induced by lipopolysaccharide (LPS) and secreted higher levels of interleukin 1-β (IL-1β) due to increased inflammasome activation in bone marrow-derived macrophages. Here, we report lower interleukin 1-β processing and caspase-11 expression in bone marrow-derived macrophages prepared from mice that have an additional copy of the stefin B gene. Increased expression of stefin B downregulated mitochondrial reactive oxygen species (ROS) generation and lowered the NLR family pyrin domain containing 3 (NLRP3) inflammasome activation in macrophages. We determined higher AMP-activated kinase phosphorylation and downregulation of mTOR activity in stefin B trisomic macrophages—macrophages with increased stefin B expression. Our study showed that increased stefin B expression downregulated mitochondrial ROS generation and increased autophagy. The present work contributes to a better understanding of the role of stefin B in regulation of autophagy and inflammasome activation in macrophages and could help to develop new treatments.

## 1. Introduction

Stefin B (cystatin B) is a type I cystatin located in the cytosol, mitochondria, and nucleus. The gene for stefin B is located on human chromosome 21 and its expression is upregulated in the brains of individuals with Down syndrome [1]. Loss-of-function mutations in the stefin B-encoding gene lead to Unverricht–Lundborg disease (progressive myoclonus epilepsy type 1, EPM1). The clinical manifestations of the disease are myoclonus and ataxia with progressive brain degeneration [2,3,4,5]. A stefin B-deficient mouse shows the pathology of EPM1, like myoclonus and progressive brain degeneration [6,7,8]. We previously showed that stefin B interacts with histones H2A.Z, H2B, and H3 and cathepsin L in the nucleus [9], and another report on murine neural cells revealed that stefin B regulated histone H3 cleavage by inhibiting cathepsins B and L [10]. Stefin B levels in macrophages were upregulated upon oxidative stress [11], and stefin B deficiency led to oxidative stress-induced apoptosis of cerebellar granule neurons [12].

In a previous publication, we showed that stefin B-deficient mice were significantly more sensitive to sepsis induced by lipopolysaccharide (LPS) and secreted higher levels of IL-1β due to increased inflammasome activation in bone marrow-derived macrophages (BMDMs) [13]. Early microglial activation and inflammation preceded the loss of neurons in the brains of mice that lacked stefin B [8], suggesting a role for stefin B in neuroinflammation [14]. Moreover, neuroinflammation was accompanied by peripheral inflammation in stefin B-deficient mice [15,16]. Bauldt et al. examined the effect of an additional copy of the stefin B-encoding gene on spontaneous epileptic activity in a stefin B trisomic mouse model and found that increased stefin B expression did not induce spontaneous epileptic activity [17].

The NLR family pyrin domain containing 3 (NLRP3) inflammasome consists of the sensor NLRP3, an adapter apoptosis-associated speck-like protein (ASC), and caspase-1; it requires a two-step process for activation [18]. Priming, the first step, is triggered by pattern recognition receptors and upregulates NLRP3 and pro-interleukin-1β (IL-1β) gene expression. The second step follows the recognition of NLRP3 activators and results in a change of conformation in NLRP3 and the assembly of an inflammasome complex consisting of ASC and caspase-1 [18]. Active caspase-1 cleaves the gasdermin D (GSDMD), a pore-forming protein, initiating pyroptosis [19,20]. Different to caspase-1, caspase-11 (caspase-4/-5 in humans) interacts with LPS in the cytosol and does not need NLRP3 activation [21,22]. Active caspase-11 cleaves GSDMD, and the cleavage results in pyroptotic cell death. However, IL-1β or IL-18 is not cleaved by caspase-11 [19,23].

Autophagy is a catabolic process that is necessary for maintaining homeostasis within the cell [24,25]. During autophagy, parts of the cytoplasm and organelles are targeted to autophagosomes and lysosomes for degradation [24]. Autophagy has a vital function in the innate immune response and NLRP3 inflammasome activation [26,27,28,29]. Serine/threonine protein kinase, a mammalian/mechanistic target of rapamycin (mTOR), is essential for the control of metabolism, proliferation, and inflammation. MTOR has only one gene in higher vertebrates, but it can form two distinct TOR complexes, mTORC1 and mTORC2. They differ in the composition of accessory proteins and in specific substrates and functions. mTORC1 regulates anabolic processes, while mTORC2 could activate pro-survival pathways [30,31].

MTORC1 inhibits autophagy by downregulating the autophagy-initiating factor UNC-51, like the kinase (Ulk1) complex [32]. We previously reported impaired autophagy flux, increased mTOR activation following LPS stimulation, and increased NLRP3 inflammasome activation in cystatin C-deficient BMDMs [33].

This study aimed to clarify the molecular mechanisms through which stefin B downregulates inflammation. On the basis of previously published work, we tested mitochondrial functions and autophagy in stefin B 3n BMDMs and mouse macrophages overexpressing stefin B.

This study provides evidence that increased stefin B expression prevents the accumulation of mitochondrial ROS and downregulates NLRP3 inflammasome activation. We demonstrated that stefin B induces autophagy via mTOR signalling. These findings highlight the role of stefin B in the regulation of autophagy and inflammasome activation in macrophages.

## 2. Materials and Methods

### 2.1. General Reagents

A list of resources used in this study is provided in Supplementary Materials and Methods (Table S1).

### 2.2. Mice

Stefin B trisomic mice were established using the C57BL/6 strain [17]. All experiments were conducted in accordance with the protocols approved by the Administration for Food Safety, Veterinary Sector, and Plant Protection, as well as the Government Ethical Committee (34401-49/2014/7 and U34401-8/2019/5). To minimise pain and suffering, mice were sacrificed via cervical dislocation. Animal care and experimental procedures were conducted in accordance with the “Guide for the Care and Use of Laboratory Animals” and with the Slovene law for animal protection published in May 2013 (“Rules on conditions for animal experiments”).

### 2.3. Preparation of Macrophages

Primary mouse BMDMs were obtained by differentiating murine bone marrow cells isolated from femurs and tibiae in the conditioned L929 medium, as described before [11]. Cells were differentiated in Dulbecco’s Modified Eagle Medium (DMEM) supplemented with 20% L929 medium, 20% foetal calf serum, 20 UI of penicillin, 20 µg/mL of streptomycin, 2 mM L-glutamine, 1% MEM non-essential amino acids, and 50 µM β-mercaptoethanol on 10 cm petri dishes in an incubator with 5% CO_2_ at 37 °C. RAW264.7 mouse macrophages were stably transfected with pcDNA3 and pcDNA/stefin B-HisTag plasmids using Lipofectamine 2000 (Invitrogen); cells were cultured in complete DMEM medium containing G418 Geneticin (Sigma Aldrich, St. Louis, MO, USA), and stable transfectants were selected, as previously described [11].

### 2.4. Quantitative Real Time PCR

Total RNA was isolated from the BMDMs using the PureLink RNA Mini Kit, following their stimulation with LPS (100 ng/mL) in DMEM for 4 h. The RNA was then treated with DNase from the TURBO DNA-*free* Kit. RNA concentrations and A_260_/A_280_ and A_260_/A_230_ ratios were determined using a NanoDrop spectrophotometer. cDNA was synthesised from the RNA using the Precision nanoScript Reverse Transcription Kit using random nanomer primers. Real-time PCR was performed on the Mx3005P qPCR system using the following thermal profile: 95 °C for 10 min followed by 40 cycles of 95 °C for 15 s and 60 °C for 1 min. The mRNA expression profiles of target genes were normalised to the expression of *Gapdh* and *B2m*; mRNA expression was calculated considering real-time PCR efficiencies using Relative Expression Software Tool 2009 and presented as a fold increase relative to unstimulated cells (control). The mRNA expression levels of the genes in the control samples were normalised to 1.0.

### 2.5. Cell Lysate Preparation and Western Blot Analysis

BMDMs were prepared as described above, seeded in a 6-well plate (2 × 10^6^ cells/well), and stimulated as indicated. For autophagy induction, cells were pretreated with Bafilomycin A1 (80 nM) and then treated with LPS (100 ng/mL) for the indicated times. Concentrations of protein lysates were assessed using Bradford assay, and equivalent amounts of proteins from cell lysates were analysed using sodium dodecyl–polyacrylamide gel electrophoresis (SDS–PAGE) followed by Western blotting, as described previously [11]. The proteins were visualised using ECL according to the manufacturer’s instructions. The signals were quantified via densitometry using ImageJ software (http://rsb.info.nih.gov/ij/index.html, accessed on 23 November 2023) according to the instructions described at https://www.unige.ch/medecine/bioimaging/files/2014/1208/6025/GelAnalysis (accessed on 21 November 2019).

### 2.6. Cathepsin Activity Measurements

To determine cathepsin activity in cell lysates, Raw 264.7 mouse macrophages were obtained from the American Type Culture Collection (ATCC TIB-71). Passages 25–29 were used in the experiments. Raw 264.7 mouse macrophages were stably transfected with stefin B (RMA cells), as reported before [11]. Cells were seeded in 24-well plates (2 × 10^5^ cells/well) and stimulated with LPS, as indicated. Negative controls were pretreated with 15 µm broad cathepsin inhibitor E-64d for 1 h. The supernatants were collected after treatment, and cells were washed with PBS. Cathepsin activity was measured as previously described [11] using fluorogenic substrates Z-ArgArg-AMC (Z-RR-AMC) and Z-PheArg-AMC (Z-FR-AMC) at a final concentration of 30 µM. The liberation of AMC was measured using a fluorimeter and normalised to the concentration of proteins in cell lysates determined using the Bradford assay. Results were normalised to the mean value of samples in the control group.

### 2.7. Cytokine Analysis

BMDMs were seeded in a 96-well plate (10^5^ cells/well) and stimulated as indicated. After treatment, the concentration of IL-1β was determined in cell culture supernatants via classical ELISA following the manufacturer’s instructions. IL-1β concentrations were normalised to protein concentrations in cell lysates determined using the Bradford assay.

### 2.8. Nitric Oxide Measurements

Nitric oxide (NO) release in BMDM supernatants was determined by measuring nitrite using the Griess reaction [34]. BMDMs were cultured in 96-well plates and stimulated with interferon-γ (IFN-γ) for 18 h, or they were IFN-γ stimulated overnight and then stimulated with LPS for 18 h. Griess solution was added to the cell culture supernatants, and absorbance was determined at 545 nm using a microplate reader (Tecan Infinite, Männedorf, Switzerland). NO concentrations were normalised to protein concentrations in cell lysates determined using the Bradford assay.

### 2.9. Cytotoxicity, Lactate Dehydrogenase Release

BMDMs were seeded in a 96-well plate (10^5^ cells/well) and treated as indicated. Release of lactate dehydrogenase (LDH) into the medium was assessed using the cytotoxicity detection kit according to the manufacturer’s instructions. Cells were lysed using 2% Triton X-100 to obtain the total LDH. Cytotoxicity was expressed as the percentage of total LDH released.

### 2.10. Flow Cytometry

Mitochondrial membrane potential (Δψ_m_) was determined using MitoTracker Red CMXRos (50 nM), and the total mitochondrial mass was determined using MitoTracker Green (50 nM). Mitochondrial ROS were determined using MitoSOX Red (5 µM) and MitoTracker Green for the whole mitochondrial mass (50 nM). All samples were analysed using a FACSCalibur flow cytometer, and fluorescence intensity plots and density plots were analysed using FlowJo software. All flow cytometry analyses were performed following live cell gating from forward and side scatter profiles.

### 2.11. Confocal Microscopy

RAW264.7 mouse macrophages and RMA cells were plated onto coverslips and either left untreated or stimulated with LPS. After the treatment, cells were washed, probed with MitoTracker Red CMXRos (100 nm) in OptiMEM for 45 min at 37 °C, and then fixed with 4% paraformaldehyde in PBS, pH 7.2, for 15 min. The membranes were permeabilised with 0.1% Triton X-100 in PBS for 10 min. Nonspecific staining was blocked with 3% BSA (Sigma-Aldrich) in PBS, pH 7.4, for 40 min. LC3 was labelled with rabbit anti LC3 antibodies (Ab 51520, Abcam, Cambridge, MA, USA). Control samples were run in the absence of primary antibodies. As secondary antibodies, cross-adsorbed goat anti-rabbit IgG antibodies, labelled with Alexa Fluor 488 and obtained from Life Technologies-Molecular Probes, were used. Finally, cells were mounted on slides with ProLong Gold Antifade Mountant (Thermo Scientific, Invitrogen, Carlsbad, CA, USA). Immunofluorescence microscopy of optical sections was performed using the confocal laser scanning microscope Leica TCS SP5 X (Leica MicroSystems, Wetzlar, Germany). The fluorophores were excited using selected lines from a tuneable white-light laser (460–670 nm) or a diode laser (405 nm). Sequential scanning was performed to minimise crosstalk between fluorophores. Leica Application Suite Advanced Fluorescence software (LAS AF, version 2.7.3.9723, Leica MicroSystems, Wetzlar, Germany) was used for image analysis.

### 2.12. Analysis of Oxygen Consumption Rate (OCR) and Extracellular Acidification Rate (ECAR) Using Seahorse XF Analyser

RAW 264.7 (R0 and RMA) cells were seeded in Seahorse XF24 cell culture microplates (75 × 10^3^ cells/well) in growth medium (DMEM with high glucose and 10% foetal bovine serum). For the twelve-hour treatment, cells were treated with LPS (100 ng/mL) or the vehicle 36 h after seeding. The growth medium was replaced with the assay medium (Seahorse XF DMEM medium supplemented with glucose (10 mM), glutamine (2 mM), and pyruvate (1 mM)) and LPS (100 ng/mL)) or the vehicle 11 h later. Cells were then incubated for an additional 60 min at 37 °C without CO_2_ before being subjected to the mito stress test. For the four-hour treatment, cells were treated with LPS (100 ng/mL) or the vehicle 48 h after seeding. The growth medium was replaced with an assay medium containing LPS (100 ng/mL) or the vehicle 3 h later. Cells were incubated for an additional 60 min at 37 °C without CO_2_ prior to the mito stress test. The mito stress test was performed using the Seahorse XFe24 Analyser (Agilent) with oligomycin (1.5 μM), carbonyl cyanide-p-trifluoromethoxyphenylhydrazone (FCCP) (1.5 μM), and rotenone (0.5 μM) + antimycin A (0.5 μM). After analysis in Seahorse, cells were lysed with 0.1% SDS in water, and the protein content was measured using the BCA protein assay. All OCR and ECAR measurements were normalised to the protein content prior to further analysis.

### 2.13. Statistical Analysis

Results are presented as mean ± SD or mean ± SEM based on the assays used. Statistical significance of the results was determined using an unpaired Student’s *t*-test. OCR and ECAR were analysed using a two-way ANOVA with Bonferroni’s test. *p* < 0.05 was considered statistically significant.

## 3. Results

### 3.1. Additional Copy of Stefin B Downregulated Caspase-11 Expression Induced by LPS and NLRP3 Inflammasome Activation

**Figure 1:**
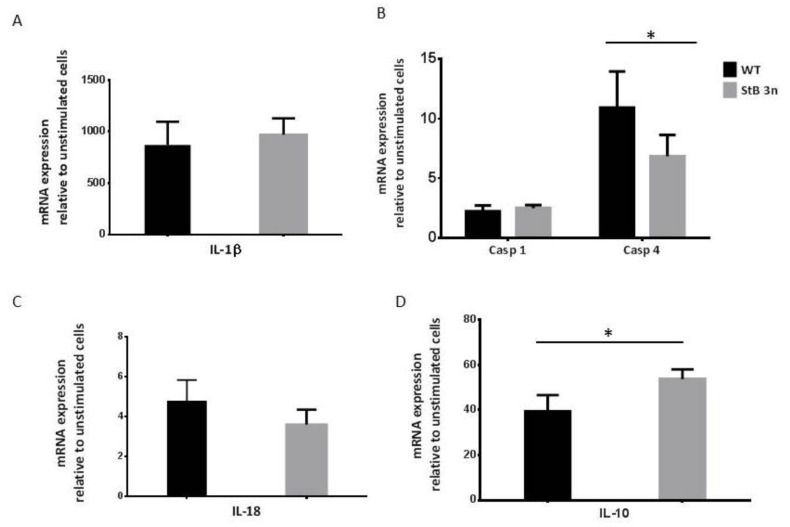
**Decreased caspase-11 mRNA levels in StB3n BMDMs following LPS stimulation.** BMDMs were challenged with LPS (100 ng/mL) for 4 h and total RNA was then isolated. (**A**–**D**) Relative mRNA expression was measured, normalised to *Gapdh* and *B2m* reference genes, and shown as fold increases compared with control (unstimulated) samples. Three independent experiments were performed in triplicate, and the results are presented as mean ± S.D. *, *p* < 0.05.

In our past study, we showed that stefin B-deficient BMDMs express higher levels of caspase-11 upon LPS challenge and had increased NLRP3 inflammasome activation [11]. To examine the effect of an additional copy of stefin B-encoding gene on pro-inflammatory gene expression, we collected BMDMs from WT and stefin B trisomic (StB3n) mice and measured the relative mRNA expression levels of caspase-1 and caspase-11 (*Casp4* gene), IL-1β, and IL-18 4 h post-LPS stimulation (Figure 1). Upon LPS stimulation, we observed upregulated *IL-1β* gene expression in both WT and StB3n BMDMs. However, the differences between the genotypes were not statistically significant (Figure 1A).

**Figure 2:**
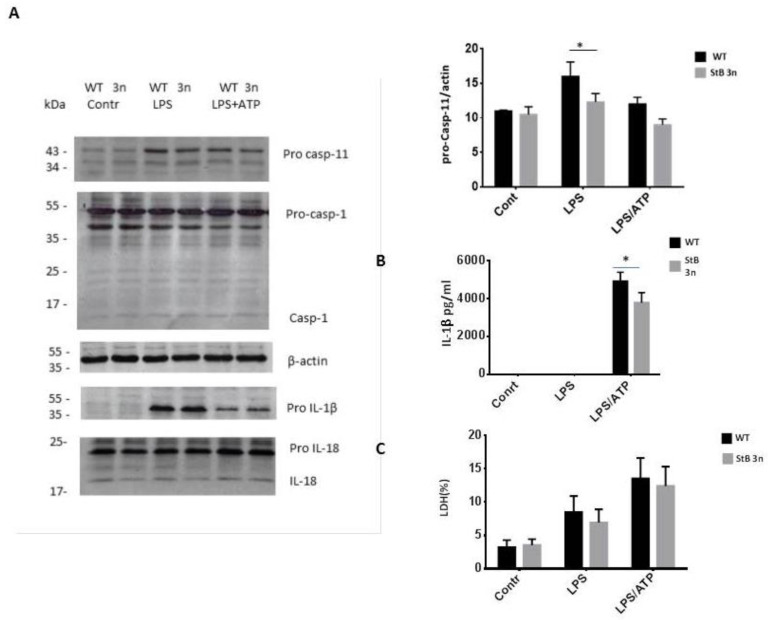
**NLPR3 inflammasome activation in StB3n BMDMs.** a(**A**) Cell lysates were blotted with the indicated antibodies. (**B**) BMDMs were seeded on 96-well plates and stimulated, as indicated. IL-1β was determined in cell culture supernatants using an enzyme-linked immunosorbent assay (ELISA). (**C**) BMDMs viability was determined using LDH release into the cell culture media. Cytotoxicity is represented as a percentage of total LDH released. Relative caspase-11, caspase-1, cleaved caspase-1, pro-IL-1*β*, pro-IL-18, and IL-18 protein band intensities were quantified using ImageJ software, and the quantities of the target proteins were normalised to those of *β*-actin. The results are representative of three independent experiments. Bars represent mean ± S.E.M. *, *p* < 0.05.

Increased stefin B expression did not influence caspase-1 expression. However, the degree of increased expression of the *Casp4* gene that encodes caspase-11 was significantly less upregulated in stefin B 3n BMDMs after LPS stimulation (Figure 1B). Regarding IL-18 mRNA, no statistically significant differences were determined between the genotypes (Figure 1C). However, we observed a significantly higher degree of increased expression IL-10 mRNA in StB3n BMDMs when compared to the level of IL-10 mRNA prepared from LPS-stimulated WT BMDMs (Figure 1D). Also, on a protein level, we determined significantly lower caspase-11 levels in StB3n BMDMs than in control cells after the LPS challenge (Figure 2A). Lower pro IL-1β cleavage was observed in LPS-/ATP-stimulated StB3n BMDMs (Figure 2A). In addition, significantly lower levels of mature IL-1β in StB3n BMDM cell cultures supernatants were determined (Figure 2B).

The degree of pyroptosis was determined by measuring the release of LDH. Although LDH levels in StB3n BMDMs supernatants reduced following stimulation with LPS/ATP, statistically significant differences between genotypes were not observed (Figure 2C). Overall, these results demonstrate reduced IL-1β secretion and caspase-11 activation in StB 3n BMDMs versus controls.

Caspase-11 activation resulted in cleavage of pannexin-1, the ATP permeable channel, and the subsequent ATP release [35], leading to activation of the purinergic P2X7 receptor, a mediator of K^+^ efflux and a crucial step in NLRP3 activation [36]. StB3n BMDMs had lower casp11 expression; thus, amplification of the NLRP3 inflammasome via caspase-11 was lower in StB3n BMDMs compared with that in control (WT) BMDMs.

### 3.2. Reduced Nitric Oxide (NO) Production in Stefin B 3n BMDMs Following IFN-γ and LPS Stimulation

**Figure 3:**
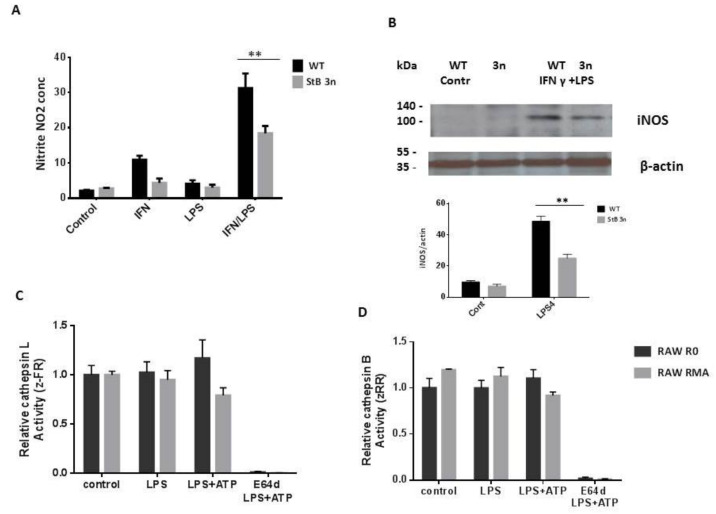
**Decreased nitric oxide (NO) production and inducible nitric oxide synthase (iNOS) expression in stefin B 3n BMDMs.** (**A**) NO release in the BMDM supernatants was measured via the Griess reagent. Macrophages were cultured in 96-well plates and stimulated with IFN-γ (100 U/mL) and LPS (100 ng/mL) for 18 h or treated overnight with IFN-γ (100 U/mL) and then stimulated with LPS (100 ng/mL) for 18 h. Absorbance was measured at 545 nm. Data were obtained from three independent experiments performed in triplicate, with three different biological samples, and the results are presented as mean ± S.D. **, *p* < 0.01. (**B**) Cells were left untreated or primed with IFN-γ (100 U/mL) and then stimulated with LPS (100 U/mL). Whole cell lysates were analysed via Western blotting with iNOS-specific antibodies. Band intensities were quantified and iNOS quantities were normalised to those of *β*-actin. The results are representative of three independent experiments. Bars represent mean ± S.E.M. **, *p* < 0.01. (**C**,**D**) Cysteine cathepsin activities in RAW 264.7 mouse macrophages and RAW 264.7 mouse macrophages stably transfected with stefin B (RMA cells). Cells were treated for 2 h with E64d (20 µM), stimulated for 4 h with LPS (100 ng/mL), and treated for 20 min with ATP (5 mM). (**C**,**D**) Cells were lysed with digitonin (200 µg/mL) and enzyme activity was measured using fluorogenic substrates: cathepsin L-like activity (zFR–AMC); cathepsin B-like activity (zRR–AMC). Three independent experiments were performed in triplicate; the results are presented as mean ± S.D. **, *p* < 0.01.

Previous studies have reported that an increase in NO levels in macrophages primed with LPS results in downregulation of ATP-induced NLRP3 inflammasome activation [37]. We further investigated whether NO was responsible for the differences in activation of NLRP3 inflammasome observed in StB3n BMDMs. We observed lower NO levels in StB3n BMDMs compared with those in WT BMDMs following stimulation with IFN-*γ*/LPS (Figure 3A), as well as lower levels of inducible nitric oxide synthase (iNOS) protein (Figure 3B), indicating that NO levels do not influence NLRP3 inflammasome activation.

In addition, we examined cytosolic cathepsin activities in control RAW 264.7 cells and RAW 264.7 cells with increased expression of stefin B (RMA cells) following NLRP3 inflammasome activation via determination of cleavage of the fluorogenic cathepsin substrates Z-FR-AMC (preferentially cleaved by cathepsin L) and Z-RR-AMC (preferentially cleaved by cathepsin B). Increased stefin B expression in RMA cells was confirmed via Western blotting (Suppl. Figure S1). Stimulation of mouse macrophages with E64d, the cathepsin inhibitor, completely inhibited cathepsin activity (Figure 3C,D). The differences in cathepsin B and L activities between the cell lines were not statistically significant (Figure 3C,D). Therefore, we concluded that cathepsin activity does not contribute to lower NLRP3 inflammasome activation.

### 3.3. Increased Stefin B Expression Prevents Accumulation of Dysfunctional Mitochondria

**Figure 4:**
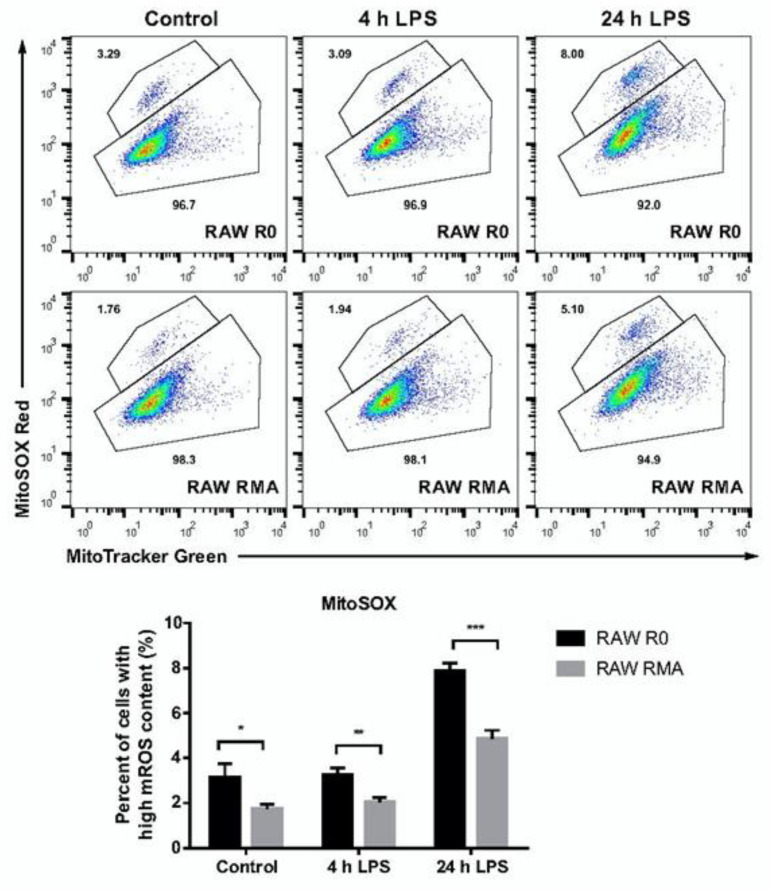
**Increased stefin B expression prevents accumulation of dysfunctional mitochondria.** RAW 264.7 mouse macrophages transfected with an empty plasmid (RAW R0 cells) or with stefin B (RAW RMA cells) were stimulated with 100 ng/mL LPS for 4 and 24 h and further labelled with MitoTracker Green (total mitochondrial mass) and MitoSOX Red (in order to determine mitochondrial ROS). Three independent experiments were performed in duplicate; the results are shown as means ± S.D. (*, *p* < 0.05, **, *p* < 0.01. ***, *p* < 0.005).

We previously demonstrated that stefin B deficiency in BMDMs leads to higher generation of mtROS [11]. In the present study, we analysed ROS-producing mitochondria using RAW264.7 mouse macrophages and RMA cells. Cells were stained with MitoTracker Green to determine the total mitochondrial content and with the MitoSOX to determine mitochondrial ROS. We observed significantly lower levels of mtROS in RAW RMA cells compared with those in control cells (RAW R0). In already unstimulated cells, the differences were even more pronounced after 4 h and 24 h of LPS stimulation (Figure 4), indicating that increased stefin B expression prevents mitochondrial ROS generation following LPS stimulation.

We previously reported that following LPS challenge, stefin B was located in the mitochondria, where it inhibited the generation of mitochondrial ROS [11]. We also reported that the increased expression of mitochondrial redox-sensitive protein peroxiredoxin 3 (Prx3) and mitochondrial superoxide dismutase 2 (Sod2) in BMDMs from the spleens of mice lacking stefin B acts as a compensation for the missing stefin B, implying a protective role for stefin B in the mitochondria [38].

### 3.4. Increased Stefin B Expression Decreases OXPHOS Activity Following LPS Treatment

**Figure 5:**
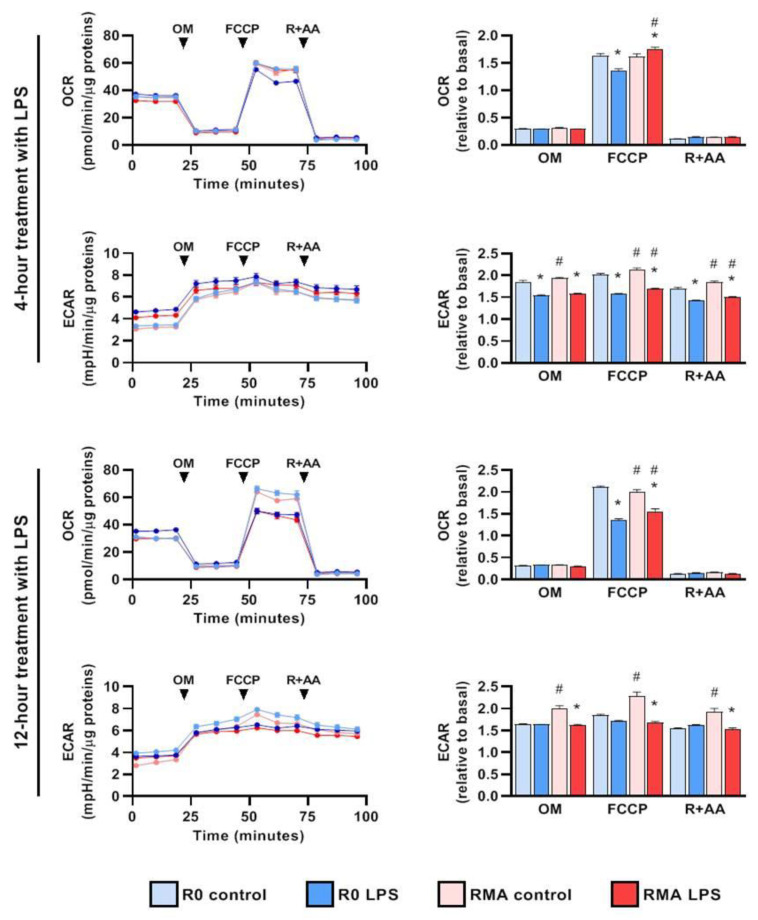
**RAW 264.7 mouse macrophages with increased expression of stefin B (RMA cells) exhibit altered metabolic profiles following LPS stimulation.** R0 and RMA RAW 264.7 cells were treated with LPS (100 ng/mL) or vehicle (control) for 4 h or 12 h. Mito stress test was then performed in Seahorse Analyzer using oligomycin (OM) (1.5 μM), FCCP (1.5 μM), and rotenone + antimycin A (R + AA) (each 0.5 μM). Graphs show group means and SEM (n = 10; 2 experiments with 5 replicates per group in each experiment). Graphs on the left: ECAR and OCR curves. Graphs on the right: OCR and ECAR after adding OM, FCCP, and R + AA, normalised to the respective basal measurement. Each data point represents the average of three consecutive normalised measurements from each well taken during each phase of mito stress. *, *p* < 0.05, R0 LPS vs. R0 control and RMA LPS vs. RMA control in the same phase of mito stress test; #, *p* < 0.05, RMA control vs R0 control and RMA LPS vs R0 LPS in the same phase of mito stress test; two-way ANOVA with Bonferroni’s test.

In mitochondria, ROS are generated during oxidative phosphorylation through leakage of single electrons from the electron transport chain [39]. We examined RAW264.7 mouse macrophages with increased stefin B expression (RMA) for changes in ECAR, measuring glycolysis and mitochondrial OCR as a measure of oxidative phosphorylation (OXPHOS) following LPS challenge. We found that OCR decreased significantly in RMA cells compared with that in controls, 4 h and 12 h after LPS stimulation (Figure 5). The ECAR, which reflects glycolytic activity in cells, was lower in unstimulated RMA cells (Figure 5). In order to determine mitochondrial functional profile real-time changes in OCR and ECAR during sequential treatment of cells with oligomycin (OM-ATP synthase inhibitor), FCCP (H^+^ ionophore) and rotenone and antimycin A (inhibitors of the electron-transport chain) were measured. We found that, compared with controls, RMA macrophages stimulated with LPS for 4 h had significantly higher absolute levels of OCR following FCCP treatment (Figure 5).

In comparison with control R0 cells, ECAR decreased in the RMA cells treated with FCCP and rotenone + antimycin following 4 h of LPS stimulation (Figure 5). ECAR increased in RMA control cells that were not treated with LPS or that were treated with LPS for 4 h (Figure 5). These results suggest that increased stefin B expression influences mitochondrial function in macrophages following LPS treatment.

### 3.5. Stefin B Promotes the Induction of Mitophagy

**Figure 6:**
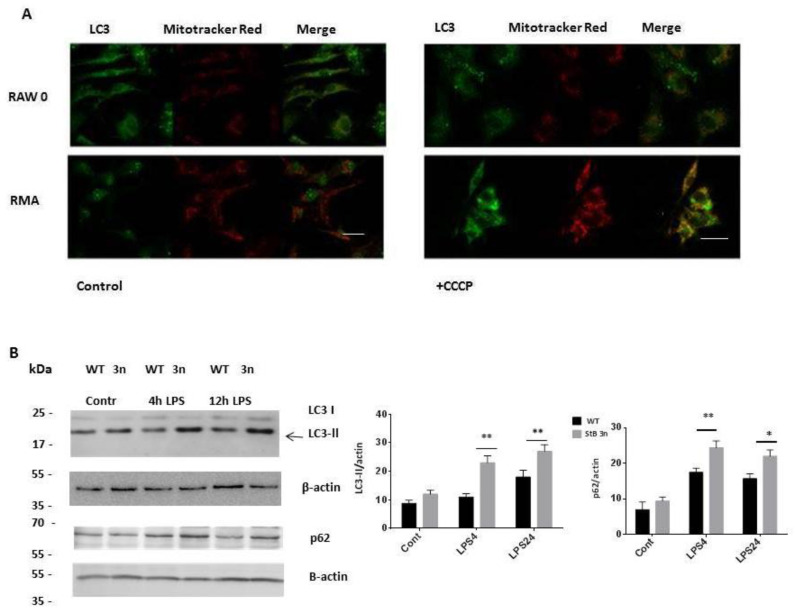
**Stefin B promotes the induction of autophagy.** (**A**) Mitochondria (red) were labelled with MitoTracker Red CMXROS, treated as written in the Materials and Methods section. Cells were further incubated with anti-LC3 antibodies (green) and labelled with Alexa conjugated secondary antibodies (Alexa 488). Co-localisation of LC3 vesicles and mitochondria was determined in merged images. The image is representative of three independent experiments. Upon carbonyl cyanide m-chlorophenylhydrazone (CCCP) treatment (20 μM for 4 h), cells were fixed and stained for LC3 and MitoTracker Red in order to evaluate mitophagy. Scale bar 10 μm. (**B**) WT and StB3n BMDMs were pretreated with Bafilomycin A1 (80 nM) for 1 h and stimulated with LPS (100 ng/mL) for the indicated times. Cell lysate proteins were analysed via immunoblotting using anti-LC3 antibodies (**A**) and P62 antibodies. Images are representative of three independent experiments. Relative LC3-II and p62 protein levels were quantified using ImageJ software and the values were normalised to those of *β*-actin. The results are representative of three independent experiments. Bars represent mean ± S.E.M. *, *p* < 0.05. **, *p* < 0.01.

We hypothesised that stefin B activates a specific autophagy process involved in the removal of damaged mitochondria following LPS challenge. It was previously reported that autophagy of human macrophages and murine RAW264.7 macrophages is induced by LPS [35]. To detect mitophagy, we assessed co-localisation of endogenous LC3 puncta and Mitotracker Red (Figure 6A).

Consistent with increased mitophagy initiation, co-localisation of LC3 with MitoTracker Red increased in RMA cells in the presence of the mitochondrial uncoupler carbonyl cyanide m-chlorophenyl hydrazine (CCCP) (Figure 6A). Notably, basal co-localisation was not significantly different between control RAW cells and RMA cells. However, upon CCCP stimulation, we observed significantly higher co-localisation of LC3 positive vesicles with MitoTracker Red in RMA cells (Figure 6A). In addition, we used in vitro autophagic flux assays in the presence of Bafilomycin A1 (Figure 6B and Figure S2) and without BafA1 (Figure S2) to analyse LC3 protein levels via Western blotting. In StB3n BMDMs pretreated with Bafilomycin A1, we observed more cleaved forms of LC3 and LC3-II compared with those in WT BMDMs, implying that StB3n BMDMs have a higher number of autophagosomes (Figure 6B). These data suggest that mitophagy, the removal of damaged mitochondria, is upregulated in StB3n BMDMs.

We previously reported impaired autophagy induction in BMDMs prepared from cystatin C-deficient mice. A lower level of LC3-II was determined in Baf1A-pretreated, LPS-stimulated, cystatin C-deficient BMDMs than in control BMDMs [33].

### 3.6. Deregulated AMPK and mTORC1 Signalling in Stefin B 3n BMDMs

**Figure 7:**
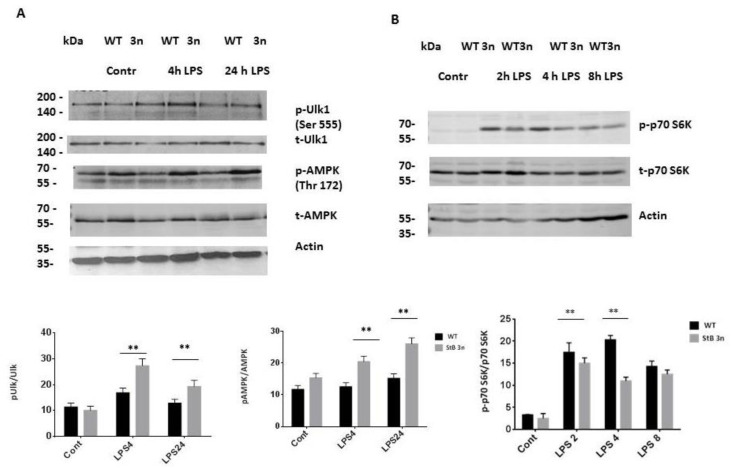
**Stefin B influences autophagy and mitochondrial integrity by inhibiting mTOR.** (**A**) BMDMs were treated with LPS (100 ng/mL) for 4 h or 24 h. (**B**) BMDMs were treated with LPS (100 ng/mL) for the indicated times. Cell lysates were immunoblotted with specific antibodies, as indicated. Images are representative of three independent experiments. Relative pUlk, pAMPK, and p-70 phosphorylated protein band intensities were quantified using ImageJ software and the values were normalised to those of *β*-actin. The results are representative of three independent experiments. Bars represent mean ± S.E.M. **, *p* < 0.01.

To further investigate the relationship between upregulated autophagy induction and altered mitophagy upon LPS stimulation in BMDMs, we examined the phosphorylation levels of Ulk1 protein, which is required not only for autophagy induction but also for mitophagy [40]. We observed no differences in total Ulk1 expression in BMDMs between the genotypes (Figure 7A), but we determined higher Ulk1 Ser-555 phosphorylation levels in StB3n BMDMs upon LPS stimulation. Ulk1 is the substrate for AMPK α1 [37,38]. Therefore, we examined whether increased Ulk1 phosphorylation is caused by AMPK activation.

We observed increased AMPK phosphorylation in StB3n BMDMs compared with that in control WT BMDMs (Figure 7A). AMPK phosphorylation suppresses mTOR activity [39]. Given the observed effects of stefin B on AMPK phosphorylation and mitophagy, we investigated the effect of increased stefin B expression on mTOR activity. Previous studies have demonstrated that LPS challenge of mouse BMDMs results in mTORC1 activation [40]. MTOR kinase activity determination was based on the phosphorylation of its substrate, the ribosomal protein S6 kinase beta-1 (p70 S6K). MTOR substrate p70 S6K phosphorylation was detected upon LPS treatment in WT and stefin B 3n BMDMs; however, it was detected to a lesser extent in StB3n BMDMs in comparison with WT BMDMs (Figure 7B). Conversely, in stefin B-deficient BMDMs, we confirmed diminished Ulk1 and AMPK phosphorylation after LPS treatment and increased mTOR signalling, as determined by the phosphorylation of its substrate, the ribosomal protein S6 kinase beta-1 (p70 S6K) (Figure S3).

Together, these results suggest that stefin B suppresses mTOR signalling. Our study reveals that stefin B inhibits mTOR signalling and that it upregulates autophagy during LPS-induced inflammation.

## 4. Discussion

Cystatins, endogenous inhibitors of lysosomal cathepsins [41,42,43], participate in regulation of innate and adaptive immune responses [44,45]. We examined the role of increased expression of the cytosolic and nuclear cystatin stefin B in autophagy and inflammasome activation. We observed reduced caspase-11 expression and activation in LPS-treated StB3n BMDMs in comparison to control cells (Figure 1) and lower IL-1β secretion in LPS-/ATP-stimulated stefin B 3n BMDMs (Figure 2), a result that is in agreement with that of our published research on inflammasome activation in stefin B-deficient BMDMs [13]. Recent studies of the secreted cystatin cystatin C revealed that the addition of cystatin C to LPS-stimulated human monocytes significantly decreased the release of IL-1β [46]. Lack of cystatin C increased caspase-11 expression in BMDMs following LPS stimulation, and cystatin C-deficient BMDMs secreted reduced IL-1β following NLRP3 inflammasome activation [33]. In addition, we observed lower NO generation in LPS-/IFN-γ-treated StB3n BMDMs (Figure 3), in line with lower NO expression in stefin B-deficient BMDMs [11].

Rinne et al. published that reduced stefin B levels correlated with higher cathepsin activity in blood cells isolated from EPM1 patients [47]. Mouse macrophages overexpressing stefin B had lower cathepsin B and L activities following NLRP3 inflammasome activation (Figure 3C,D). These results are in line with the published observations that StB-deficient BMDMs have increased cathepsin B and L activities. However, cathepsin activities were not linked to activation of NLRP3 inflammasome in stefin B-deficient BMDMs [13].

Several studies have implicated the role of ROS derived from mitochondria in activation of NLRP3 inflammasome. We previously reported that stefin B KO BMDMs have increased mtROS levels following LPS stimulation [11]. Conversely, we observed a decreased number of ROS-producing mitochondria and increased mitophagy in LPS-stimulated RAW 264.7 mouse macrophages stably transfected with stefin B (RMA cells) (Figure 4 and Figure 6A). Interestingly, results from a proteomics study of cerebellar synaptosomes prepared from StB-deficient mice revealed significant changes in the nuclear DNA-encoded mitochondrial proteome [48]. Furthermore, another study reported defective mitochondrial respiration in differentiating stefin B-deficient neural cells and confirmed downregulation of genes that encode proteins in the electron transport chain [10]. Neuronal cell differentiation resulted in an increase in the oxygen consumption rate of neuronal precursors, but it was delayed in stefin B-deficient cells [10]. We studied mitochondrial respiration and found that upon LPS stimulation, OXPHOS decreased in RMA cells compared with that in control RAW 264.7 macrophages. Interestingly, a study of LPS-stimulated IL-10-deficient BMDMs revealed that IL-10 inhibited glycolysis and promoted OXPHOS [49]. In addition, IL-10 suppressed mTOR activity and promoted the elimination of damaged mitochondria [49]. We previously demonstrated lower expression of IL-10 in BMDMs prepared from stefin B-deficient mice [11], while IL-10 expression in StB3n BMDMs increased following LPS stimulation when compared to WT control BMDMs (Figure 1D). Higher IL-10 levels determined in macrophages from StB3n mice may participate in downregulation of mTOR activation in stefin B 3nBMDMs, compared with that in WT BMDMs.

In inflammatory responses, autophagy has a protective role downregulating the inflammation by removing damaged ROS-producing mitochondria [27,50]. Several studies reported the effect of mitophagy in the activation of NLRP3 inflammasome [49,51,52]. We observed increased LC3 II cleavage and higher levels of the autophagy adaptor protein SQSTM1/p62 in StB3n BMDMs (Figure 4B). Karin’s group reported that SQSTM1/p62 plays a vital role in mitophagy upon activation of NLRP3 inflammasome [51]. Moreover, it was reported that Sestrin 2, a leucine sensor of the mTOR pathway [53], induces mitophagy and suppresses NLRP3 inflammasome activation in macrophages [52]. Sestrin 2 activates mitophagy via upregulation of the Ulk1 protein [52]; Ulk1-deficient mice had defective mitophagy and accumulated damaged mitochondria [54]. We did not determine changes in Ulk1 protein levels in StB3n BMDMs, but there was an increase in Ulk1 Ser-555 phosphorylation (Figure 7). We observed increased AMPK activation and lower mTOR activation in StB3n BMDMs than in WT control cells, as determined from the phosphorylation of its substrate, S6 kinase beta-1 (p70 S6K) (Figure 7). Complementary experiments in stefin B-deficient BMDMs showed diminished Ulk1 and AMPK phosphorylation and increased mTOR signalling, as determined from the phosphorylation of its substrate, the ribosomal protein S6 kinase beta-1 (p70 S6K) (Figure S3). In stefin B-deficient mice, we reported increased NLRP3 activation and mortality upon LPS-induced sepsis [13].

Li et al. showed that inhibiting mTOR signalling impaired activation of NLRP3 inflammasome in macrophages in vitro [55]. Published studies reported that autophagy is induced by cystatin C either through AMPK activation or by inhibiting mTOR activity [56,57,58,59]. AMPK, when phosphorylated, suppresses mTOR activity [60]. We previously reported lower levels of AMPK phosphorylation and increased mTOR activity in cystatin C-deficient BMDMs following LPS stimulation [33]. Sabatini et al. recently showed that AMPK and HRI kinases mediate the inhibition of mTORC1 due to mitochondrial dysfunction [61]. It was demonstrated that stefin B deficiency impairs mitochondrial function [10,48]. We propose that stefin B protects mitochondria from damage, and the increased AMPK activation determined in StB 3nBMDMs reduces mTOR activation [55].

Although the two cystatins stefin B and cystatin C have different inhibitory profiles and localisation within the cell, both inhibit the mTOR signalling. Kaur et al. reported that EPM1 symptoms in stefin B-deficient mice were alleviated by cystatin C overexpression and were conversely exacerbated by cystatin C deficiency [62]. It is tempting to speculate that the reversal of some pathological changes in stefin B-deficient mice via increased cystatin C expression could be due to its effect on the AMPK/mTOR pathway. Our study demonstrated that stefin B is a negative regulator of mTOR signalling during LPS-induced inflammation. This observation broadens the functions of this inhibitor in the context of health and disease.

## 5. Conclusions

We showed that increased expression of stefin B induces autophagy and diminishes the inflammatory response. In addition, it prevents mitochondrial ROS formation and impairs NLRP3 inflammasome activation. Stefin B confers anti-inflammatory effects via the AMPK/mTOR signalling pathway. These results contribute to a better understanding of cystatins, specifically stefin B, in the regulation of autophagy and inflammasome activation in macrophages.

## Data Availability

All data generated or analysed during this study are included in this published article.

## Supplementary Materials

The following supporting information can be downloaded at https://www.mdpi.com/article/10.3390/cells12232731/s1. Table S1: List of resources used in this study. Figure S1: Stefin B In R0 and RMA cells. Figure S2: Stefin B enchance the induction of autophagy. Figure S3: Stefin B deficiency influences AMPK and mTOR signalling [63].
