# Supplementary material for: Stefin B Inhibits NLRP3 Inflammasome Activation via AMPK/mTOR Signalling
Source: Cells. 2023 Nov 29;12(23):2731. doi: 10.3390/cells12232731 (PMC10798374; doi:10.3390/cells12232731)
Supplement: Supplementary file 1 [file cells-12-02731-s001.zip › cells-2735324-supplementary.pdf]

cells MDPI

Article

Stefin B Inhibits NLRP3 Inflammasome Activation via AMPK/mTOR Signalling

Mojca Trstenjak-Prebanda [1], Monika Biasizzo [1,2], Klemen Dolinar [3], Sergej Pirkmajer [3], Boris Turk [1,4], Veronique Brault [5], Yann Herault [5,6] and Nataša Kopitar-Jerala [1,*]

[1] Department of Biochemistry, Molecular and Structural Biology, Jožef Stefan Institute, SI-1000 Ljubljana, Slovenia; mojca.prebanda@ijs.si (M.T.-P.); monika.biasizzo@ijs.si (M.B.); boris.turk@ijs.si (B.T.)
[2] International Postgraduate School Jožef Stefan, SI-1000 Ljubljana, Slovenia
[3] Institute of Pathophysiology, Faculty of Medicine, University of Ljubljana, SI-1000 Ljubljana, Slovenia; klemen.dolinar@mf.uni-lj.si (K.D.); sergej.pirkmajer@mf.uni-lj.si (S.P.)
[4] Faculty of Chemistry and Chemical Technology, University of Ljubljana, SI-1000 Ljubljana, Slovenia
[5] Institut de Génétique et de Biologie Moléculaire et Cellulaire (IGBMC), INSERM, CNRS, Université de Strasbourg, 1 rue Laurent Fries, 67404 Illkirch Graffenstaden, France; vbrault@igbmc.fr (V.B.); herault@igbmc.fr (Y.H.)
[6] Institut Clinique de la Souris, PHENOMIN, CELPHEDIA, INSERM, CNRS, Universite' de Strasbourg, 67404 Illkirch Graffenstaden, France
* Correspondence: natasa.kopitar@ijs.si; Tel.: +386-1-477-3510

Supplementary Materials and Methods

General Reagents

Table S1. List of resources used in this study.

Reagent or resource	Source	Identifier
**Antibodies and recombinant proteins**		
mouse monoclonal anti-caspase-1	Novus Biologicals LLC, Centennial, CO, USA	Cat# NB100-56565
rat monoclonal anti-caspase-11	Abcam, Cambridge, MA, USA	Cat# ab10454
mouse monoclonal anti-IL-1β	National Cancer Institute, Frederick, MD, USA	3ZD
rabbit polyclonal anti-IL-18	Abcam, Cambridge, MA, USA	Cat# ab71495
rabbit polyclonal anti-iNOS	Abcam, Cambridge, MA, USA	Cat# ab3523
rabbit polyclonal anti-mTOR phospho S2448	Invitrogen, Carlsbad, CA, USA	Cat# 441125G
rabbit polyclonal anti-mTOR	Cell Signalling Technology, Inc., Danvers, MA	Cat# 2972
rabbit monoclonal anti-p70 S6 kinase phospho T389	Cell Signalling Technology, Inc., Danvers, MA	Cat# 9234
rabbit monoclonal anti-p70 S6 kinase	Cell Signalling Technology, Inc., Danvers, MA	Cat# 2708
rabbit monoclonal anti-phospho-AMPKα (Thr172)	Cell Signalling Technology, Inc., Danvers, MA	Cat# 2535
mouse monoclonal anti-AMPKα1/2	Santa Cruz Biotechnology, Inc., Dallas, TX, USA	Cat# sc-74461

rabbit polyclonal anti-LC3B	Abcam, Cambridge, MA, USA	Cat# ab51520
mouse monoclonal anti-p62	Santa Cruz,	Cat# sc-48402
mouse monoclonal anti-β-actin	Sigma-Aldrich, St. Louise, MO, USA	Cat# A1978
Rabbit polyclonal Ulk1	Cell Signalling Technology, Inc., Danvers, MA	#8359
rabbit monoclonal anti-phospho-Ulk Ser555	Abcam, Cambridge, MA, USA	ab133747
Mouse monoclonal anti stefin B antibodies	(Kopitar-Jerala et al., 1993)	A6/2
**Reagents, Buffers, and Solutions**		
DMEM	Sigma-Aldrich, St. Louise, MO, USA	Cat# D6429
FBS Heat-Inactivated	Sigma-Aldrich, St. Louise, MO, USA	Cat# F9665
DPBS	BioWest, Nuaillé, France	Cat# L0625
Penicillin-Streptomycin	Sigma-Aldrich, St. Louise, MO, USA	Cat# P4333
L-Glutamine solution	Sigma-Aldrich, St. Louise, MO, USA	Cat# G7513
MEM Non-Essential Amino Acids Solution (100X)	Gibco (Life Technologies), Paisley, UK	Cat# 11140035
D-(+)-glucose	Riedel-de-Haën	Cat# G8270
Sodium pyruvate solution	Sigma-Aldrich, St. Louise, MO, USA	Cat# S8636
β-mercaptoethanol	Sigma-Aldrich, St. Louise, MO, USA	Cat# 805740
OptiMEM	Invitrogen, Carlsbad, CA, USA	Cat# 31985-062
Tryple Select	Gibco (Life Technologies), Paisley, UK	Cat# 12563-029
LPS (E. coli 055:B5)	Sigma-Aldrich, St. Louise, MO, USA	Cat# L6529
ATP	Sigma-Aldrich, St. Louise, MO, USA	Cat# A6419
G418 (Geneticin)	Sigma-Aldrich, St. Louise, MO, USA	Cat# # G8168
CCCP	Sigma-Aldrich, St. Louise, MO, USA	Cat# C2759
Bafilomycin A1	Cayman Chemical, Ann Arbor, MI, USA	Cat# 11038
FCCP	Sigma-Aldrich, St. Louise, MO, USA	Cat# C2920
Antimycin A	Sigma-Aldrich, St. Louise, MO, USA	Cat# A8674
Oligomycin A	Merck Millipore, Burlington, MA, USA	Cat# 495455
Rotenone	Merck Millipore,	Cat# 557368

	Burlington, MA, USA	
DOTAP	Roche Applied Science, Penzberg, Germany	Cat# 11202375001
E-64d	Peptide Institute, Osaka, Japan	Cat# 4321-v
Ca-074 me	MedChemExpress, Monmouth Junction, NJ, USA	Cat# HY-100350
z-FR-AMC	Bachem, Bubendorf, Switzerland	Cat# I-1160
z-RR-AMC	Bachem, Bubendorf, Switzerland	Cat# I-1135
Digitonin	Sigma-Aldrich, St. Louise, MO, USA	Cat# D141
Complete protease inhibitor mixture	Sigma-Aldrich, St. Louise, MO, USA	Cat# P8340
Phosphatase arrest cocktail	G-Biosciences, St. Louis, MO	Cat# 786-647
**Commercial assays**		
PureLink RNA Mini Kit	Ambion, Austin, TX, USA	Cat# 12183018A
TURBO DNA-*free* Kit	Ambion, Austin, TX, USA	Cat# AM1907
Precision nanoScript Reverse Transcription Kit	Primerdesign Ltd., Chandler's Ford, UK	Cat# RT-nanoScript
mouse geNorm Reference Gene Selection Kit	Primerdesign Ltd., Chandler's Ford, UK	Cat# ge-DD-12-mo
mouse IL-1 beta ELISA Ready-SET-Go!	eBioscience Technology, San Diego, CA, United States	Cat# 88-7013-22
Cytotoxicity Detection KitPlus (LDH)	Roche Applied Science, Penzberg, Germany	Cat# 4744934001
**Equipment**		
NanoDrop 1000 Spectrophotometer	ThermoFisher Scientific, Waltham, MA, USA	N/A
Mx3005P qPCR system	Agilent, Santa Clara, CA, USA	N/A
G:BOX Chemi XR	Syngene,	N/A
Tecan Infinite M1000 Pro	Tecan, Gröding, Austria	N/A
FACSCalibur flow cytometer	Becton Dickinson, Franklin Lakes, NJ, USA	N/A
**Software and algorithms**		
REST 2009 (Relative Expression Software Tool)	Technical University Munich, Munich, Germany QIAGEN, Hilden, Germany	N/A
GraphPad Prism	GraphPad Software, San Diego, CA, USA	N/A
GeneSys	Syngene, Cambridge, UK	N/A

CellQuest Pro	Becton Dickinson, Franklin Lakes, NJ, USA	N/A
FlowJo v10	FlowJo LLC., Ashland, OR, USA	N/A

Primers for quantitative real-time PCR.

Protein (gene)	Forward Primer	Reverse Primer
IL-1β (*Il1b*)	GCTATGGCAACTGTT CCTGAA	ACAGCCCAGGTCAAA GGTT
IL-18 (*Il18*)	CCAAGTTCTCTTCGT TGACAAAA	GTCCTCTTACTTCACT GTCTTTG
Caspase-1 (*Casp1*)	CTGCGGTGTAGAAA AGAAACG	TCCATTTATTGTCCCT ATACTCACT
Caspase-11 (*Casp4*)	GCTACGATGTGGTG GTGAAA	GGAATGTGCTGTCTG ATGTCT

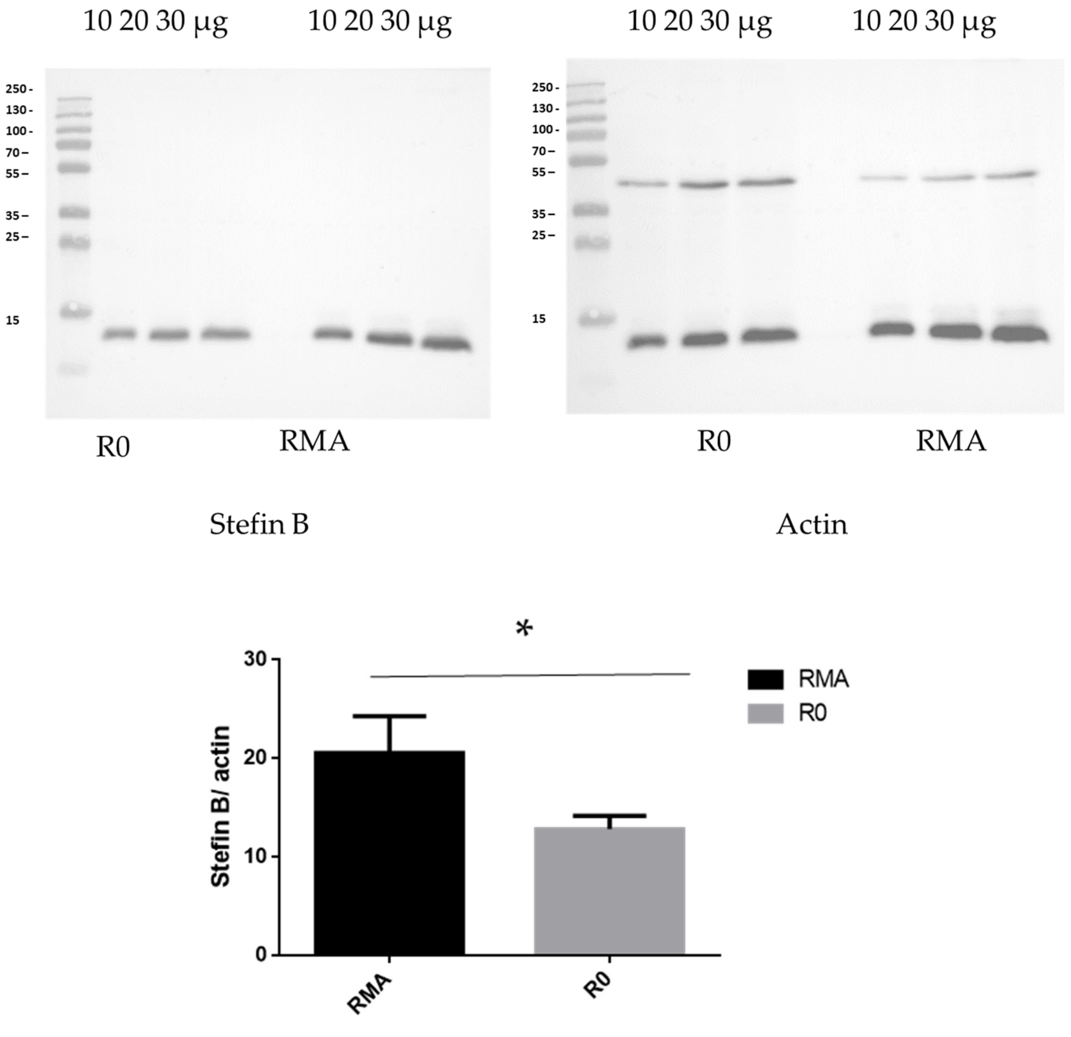

Supplementary Figure S1. Stefin B In R0 and RMA cells. RO and RMA cells were 10, 20, 30 µg cell lysate were analyzed by Western blot using anti-stefin B antibodies. Band intensities were quantified and stefin B quantities were normalised to that of *β*-actin. Bars represent mean ± S.E.M. *, p<0.01.

Cell Lysate Preparation and Western Blot Analysis

BMDMs were prepared as described above, seeded in a 6-well plate (2x10^6 cells/well), Concentrations of protein lysates were determined using Bradford assay and 10,20 and 30 µg of proteins from cell lysates were analysed using sodium dodecyl–polyacrylamide gel electrophoresis (SDS–PAGE) followed by western blotting with stefin B specific monoclonal antibodies, as described previously [63]. The proteins were visualised using ECL according to the manufacturer's instructions. The signals were quantified via densitometry using ImageJ software (http://rsb.info.nih.gov/ij/index.html) according to the instructions described at https://www.unige.ch/medecine/bioimaging/files/2014/1208/6025/GelAnalysis (accessed on 21 November 2019).

References :

63. Kopitar-Jerala, N.; Curin-Serbec, V.; Jerala, R.; Krizaj, I.; Gubensek, F.; Turk, V. Monoclonal antibodies to human stefin B and determination of their epitopes. *Biochimica et Biophysica Acta* 1993, *1164*, 75-80.

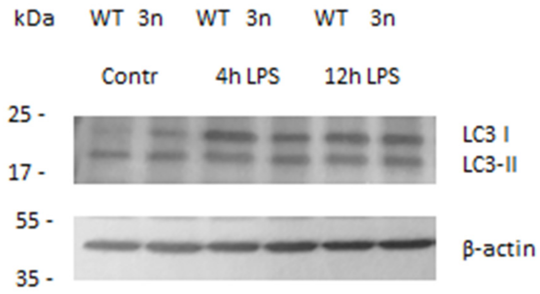

Supplementary Figure S2: Stefin B enchance the induction of autophagy. WT and stefin B 3n BMDMs were stimulated with LPS (100 ng/mL) for indicated times. Cell lysates were Western blotted with indicated antibodies.

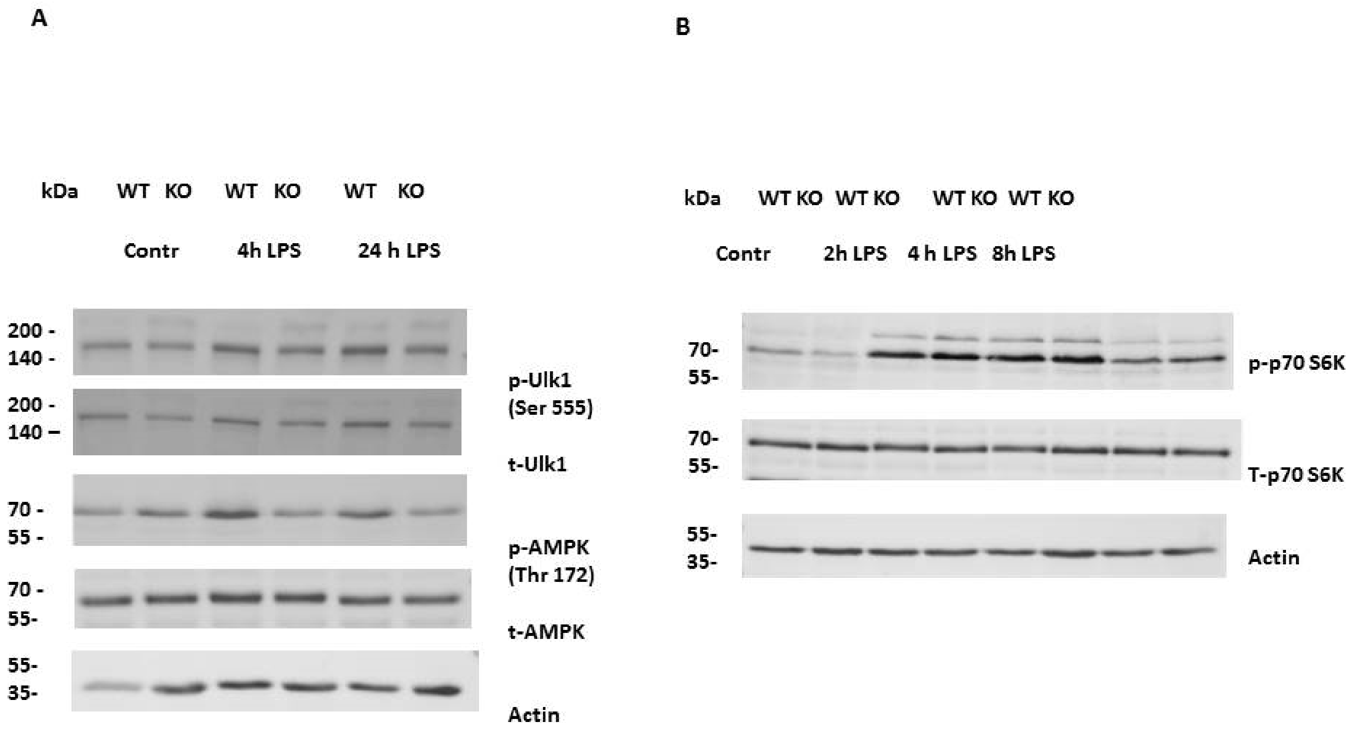

Supplementary Figure S3: Stefin B deficiency influences AMPK and mTOR signalling. A, Stefin B KO and WT BMDMs were stimulated with LPS (100 ng/ml) for 4 h or 24 h. B, Stefin B KO and WT BMDMs were stimulated with LPS (100 ng/ml) for the indicated times. Cell lysates were subjected to Western blotting with specific antibodies, as indicated.
